# Molecular detection and genetic characterization of *Bartonella* species from rodents and their associated ectoparasites from northern Tanzania

**DOI:** 10.1371/journal.pone.0223667

**Published:** 2019-10-15

**Authors:** Ndyetabura O. Theonest, Ryan W. Carter, Nelson Amani, Siân L. Doherty, Ephrasia Hugho, Julius D. Keyyu, Barbara K. Mable, Gabriel M. Shirima, Rigobert Tarimo, Kate M. Thomas, Daniel T. Haydon, Joram J. Buza, Kathryn J. Allan, Jo E. B. Halliday

**Affiliations:** 1 School of Life Sciences and Bioengineering, Nelson Mandela African Institution of Science and Technology, Arusha, Tanzania; 2 Kilimanjaro Clinical Research Institute, Moshi, Tanzania; 3 The Boyd Orr Centre for Population and Ecosystem Health, Institute of Biodiversity Animal Health and Comparative Medicine, College of Medical Veterinary and Life Sciences, University of Glasgow, Glasgow, Scotland, United Kingdom; 4 Tanzania Wildlife Research Institute, Arusha, Tanzania; 5 Centre for International Health, Dunedin School of Medicine, University of Otago, Dunedin, New Zealand; Defense Threat Reduction Agency, UNITED STATES

## Abstract

**Background:**

Bartonellae are intracellular bacteria, which can cause persistent bacteraemia in humans and a variety of animals. Several rodent-associated *Bartonella* species are human pathogens but data on their global distribution and epidemiology are limited. The aims of the study were to: 1) determine the prevalence of *Bartonella* infection in rodents and fleas; 2) identify risk factors for *Bartonella* infection in rodents; and 3) characterize the *Bartonella* genotypes present in these rodent and flea populations.

**Methods and results:**

Spleen samples collected from 381 rodents representing six different species were tested for the presence of *Bartonella* DNA, which was detected in 57 individuals (15.0%; 95% CI 11.3–18.5), of three rodent species (*Rattus rattus* n = 54, *Mastomys natalensis* n = 2 and *Paraxerus flavovottis* n = 1) using a qPCR targeting the *ssr*A gene. Considering *R*. *rattus* individuals only, risk factor analysis indicated that *Bartonella* infection was more likely in reproductively mature as compared to immature individuals (OR = 3.42, *p* <0.001). *Bartonella* DNA was also detected in 53 of 193 *Xenopsylla cheopis* fleas (27.5%: 95% CI 21.3–34.3) collected from *R*.*rattus* individuals. Analysis of *ssr*A and *glt*A sequences from rodent spleens and *ssr*A sequences from fleas identified multiple genotypes closely related (≥ 97% similar) to several known or suspected zoonotic *Bartonella* species, including *B*. *tribocorum*, *B*. *rochalimae*, *B*. *elizabethae* and *B*. *quintana*.

**Conclusions:**

The *ssr*A and *glt*A sequences obtained from rodent spleens and *ssr*A sequences obtained from fleas reveal the presence of a diverse set of *Bartonella* genotypes and increase our understanding of the bartonellae present in Tanzanian. Further studies are needed to fully characterise the prevalence, genotypes and diversity of *Bartonella* in different host populations and their potential impacts on human health.

## Introduction

*Bartonella* are fastidious, Gram-negative, vector-borne bacteria with worldwide distribution. *Bartonella* species are known to infect mainly erythrocytes and endothelial cells of various mammals, such as humans, cats, dogs, ruminants, wild rabbits and rodents [1]. Epidemiological studies have demonstrated that rodents and other small mammals are important hosts of *Bartonella* species and that ectoparasites such as fleas, ticks, sand flies, and lice are key vectors of *Bartonella* infection [2,3].

In recent years, an increasing number of *Bartonella* species have been identified as zoonotic pathogens. To date there are roughly 45 *Bartonella* species and subspecies that have been designated [4], of which at least 20 are rodent-associated [1]. Several studies indicate that rodent-associated *Bartonella* are the cause of human infections in various regions of the world, particularly in areas where humans are in close contact with rodents [5–10]. However, knowledge of the distribution and epidemiology of *Bartonella* in rodents and of the role of *Bartonella* species in human disease in Tanzania is limited.

Clinical manifestations of *Bartonella* infection in humans can range from mild [7] to life threatening disease and can present as acute or chronic [5,11]. Known sequelae attributed to *Bartonella* species include endocarditis [8], myocarditis [12], fever and neurologic disorders [13], intraocular neuroretinitis [14], meningitis, splenomegaly and lymphadenopathy [15]. This range of non-specific and variable symptoms makes *Bartonella* infections hard to diagnose clinically. This contributes to a poor understanding of the current distribution and relative importance of infections caused by this pathogen. The challenges of identifying the causes of non-specific febrile illness are demonstrated by previous research conducted in Moshi, northern Tanzania, where a study of patients admitted to hospital with febrile illness revealed that a range of zoonotic pathogens were responsible for roughly a quarter of the hospital admissions [16]. However, no zoonotic infections were included in the admission differential diagnosis for any patient in that study, indicating lack of awareness and diagnostic capacity for many zoonotic pathogens. Over the past few decades, numerous reports of bartonellosis in febrile humans have been made globally [6,8–10,17,18]. However, in developing countries bartonellosis is often not considered as a potential diagnosis.

Molecular detection and typing methods for *Bartonella* are widely used due to their greater sensitivity and ease of use in comparison to culture and serology based approaches. Real-time PCR assays are recommended for primary screening of *Bartonella* species followed by confirmatory assays, using either conventional or real-time PCR and sequencing [19,20]. In Africa, studies conducted in Ethiopia [21], Kenya [22], South Africa [23], the Democratic Republic of Congo [24,25] and Uganda [26] have previously confirmed detection of *Bartonella* species in rodents using conventional PCR detection methods for multiple gene targets. In northern Tanzania, a study performed in Mbulu, a rural district in northern Tanzania, detected *Bartonella* in 41% of indigenous rodents using *glt*A and *rpo*B PCR targets [25]. Analyses of *glt*A sequences from these samples revealed the presence of multiple genotypes similar to known *Bartonella* species, including *B*. *elizabethae*, *B*. *tribocorum*, *B*. *birtlesii* and *B*. *queenslandensis* [25].

*Bartonella* species are present in Tanzania and may contribute to the burden of human febrile illness in northern Tanzania. However, the distribution and epidemiology of *Bartonella* in Tanzania is largely unknown. The aims of this study were to use molecular diagnostic tools to estimate the prevalence of *Bartonella* infection in rodents and their fleas sampled in northern Tanzania. Specifically, we aimed to: 1) determine the prevalence of *Bartonella* infection in rodents and fleas using quantitative real-time polymerase chain reaction (qPCR); 2) identify risk factors for *Bartonella* infection in rodents; and 3) use sequencing of the *glt*A and *ssr*A genes to characterize the *Bartonella* genotypes present in these rodent and flea populations.

## Methods

### Ethics statement

Ethical approval for the study was granted by the Tanzania Commission for Science and Technology (COSTECH 2012-471-ER-2005-141 & 2015-71-NA-2011-199); Kilimanjaro Christian Medical Centre (KCMC) Ethics Committee (535 & 537); National Institute of Medical Research (NIMR), Tanzania (NIMR/HQ/R.8a/Vol.IX/1499 & NIMR/HQ/R.8a/Vol.IX/1522); Tanzania Wildlife Research Institute (TAWIRI); University of Glasgow College of Medical, Veterinary and Life Sciences Ethics Committee (200120020), and University of Glasgow Faculty of Veterinary Medicine Ethics and Welfare Committee (01a/13 & 02a/13). Written consent for study participation was obtained for each participating household, using forms translated into Swahili. Rodent sampling was performed in accordance with the UK Guidance on the Operation of the Animals (Scientific Procedures) Act 1986 and American Veterinary Medical Association Guidelines for the Euthanasia of Animals [27,28].

### Rodent trapping and sampling

**Fig 1:**
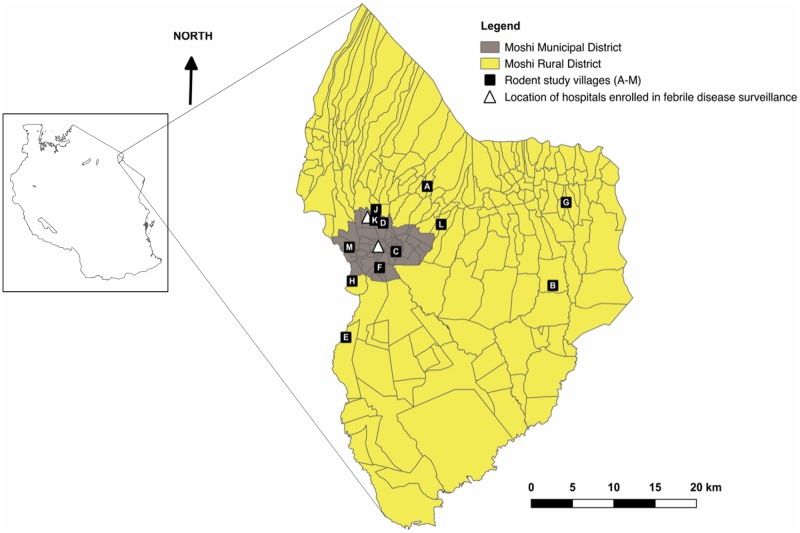
Map of Moshi Municipal and Moshi Rural Districts, showing representative locations of rodent study villages in relation to the two hospitals (Kilimanjaro Christian Medical Centre and Mawenzi Regional Referral Hospital) at which febrile illness surveillance has been conducted in previous studies. Letters indicate the different villages in which rodent trapping was conducted. Polygons in the main image show local administrative boundaries. Insert map on left shows outline of Tanzania and location of study districts within the country. This figure is adapted from a version published previously [29].

Rodent spleen samples and ectoparasites were obtained from a cross-sectional study conducted to explore the role of rodents in the epidemiology of leptospirosis and other zoonoses in the Kilimanjaro region of northern Tanzania [29]. Rodents were trapped in five villages within Moshi Municipal District and seven villages within Moshi Rural District, as previously described [29] (Fig 1). The target sample size was 50 rodents per village to give sufficient power (α = 0.95, β = 0.8) to detect a minimum infection prevalence of 10% [29]. Villages for sampling were randomly selected from a list, home to people that had sought care, and had been enrolled in previous febrile illness surveillance studies at local hospitals [16]. Rodent trapping was performed in three sessions: 1) May-June 2013 (wet season); 2) May-June 2014 (wet season); and 3) August-September 2014 (dry season). Rodents were trapped in households in a total of 12 villages through cross-sectional visits, with one additional round of repeat sampling conducted in one village (based on high trap success in the initial visit) [29]. Trapped rodents were euthanized by terminal halothane anaesthesia and cervical dislocation. Data gathered for every trapped rodent included: species (determined by observation of phenotypic characteristics and measurement of morphometric features), sex and reproductive maturity status (mature or immature determined based on external sexual characteristics [30]). A full necropsy and tissue sampling were performed for each rodent sampled. A fresh sterile scalpel blade was used for each rodent and all other necropsy equipment was washed using 5% Virkon and dried between usages to avoid cross-contamination. Spleen tissue samples were collected into sterile Eppendorf tubes and stored at -80°C prior to DNA extraction. Ectoparasites observed on trapped rodents were collected and stored in 70–96% ethanol; all ectoparasites from the same rodent were stored together. Collected fleas were identified to species level using a dissecting microscope and a pictorial flea identification guide [31]. *Xenopsylla cheopis* fleas were selected for DNA extraction and *Bartonella* testing based on their known contribution to *Bartonella* transmission [32]. For each rodent with at least one *X*. *cheopis* collected, DNA was extracted from one (if only one *X*. *cheopis* present on that host) or two (if more than one *X*. *cheopis* present on that host). Where multiple *X*. *cheopis* were collected from the same rodent, selection of individual fleas for DNA extraction was opportunistic.

### DNA extraction

DNA was extracted from approximately 10 milligrams (mg) of spleen tissue using the DNeasy Blood and Tissue Kit spin-column protocol for DNA purification from tissues (Qiagen, Hilden, Germany). DNA from spleen tissues was eluted in 100μl of AE buffer and quantified using a Nano-Drop spectrophotometer (Thermo Scientific, Waltham, MA, USA). DNA from individual whole fleas was also extracted using the DNeasy Blood and Tissue Kit, following the protocol for purification of total DNA from ticks and eluted in a final volume of 65μl AE buffer. For all extractions, a no-template extraction control (PCR-grade water) was included for every 20 samples and DNA extracts were stored at -20°C prior to testing. DNA extracts from spleens were diluted in 20μl of AE buffer to a standard DNA concentration of 10–50 ng/μl for PCR testing, to minimize the potential for PCR inhibition due to the high concentration of host DNA in the rodent spleen extracts. Due to the lower concentration of DNA in extracts from fleas, these were tested directly from extraction concentrations.

### Quantitative real-time PCR for detection of *Bartonella* species *ssr*A gene

DNA extracts from rodent spleens and fleas were screened using a *Bartonella* genus-specific real-time PCR assay (qPCR) targeting the transfer-mRNA *ssr*A gene, using a previously published protocol [20]. The original paper describing this assay reports a lower limit of detection of < 5 fg of *Bartonella* DNA, equivalent to < 3 genomic copies per reaction when tested against four *Bartonella* species (*B*. *quintana*, *B*. *henselae*, *B*. *bovis*, and *B*. *elizabethae*) [20]. The primers *ssr*A-F (5ˈ-GCTATGGTAATAAATGGACAATGAAATAA-3ˈ), *ssr*A-R (5ˈ GCTTCTGTTGCCAGGTG-3ˈ) and 6-carboxyfluorescein FAM-labelled probe (5ˈ-ACCCCGCTTAAACCTGCGACG-3ˈ-BHQ1) were used to amplify an *ssr*A gene fragment of approximately 300bp. qPCR reactions were carried out in 20 μl volumes comprised of 10 μl QuantiNova Probe PCR mix (Qiagen), 0.8 μl of each primer (10 μM) and probe (5 μM), 2.6 μl nuclease-free water and 5 μl DNA template. Positive control (rodent tissue DNA extract obtained from a previous study [24] positive for *Bartonella* with closest similarity to *B*. *tribocorum*), extraction controls and no-template controls were included in each qPCR run. Assays were performed on a Rotor-Gene Q/6000 (Qiagen) with manufacturer recommended thermocycling conditions as follows: 95 °C for 2 minutes, followed by 45 cycles of 95 °C for 5 seconds and 60 °C for 5 seconds. A qPCR run was considered valid when all negative controls showed no evidence of amplification and the positive controls amplified with a Ct value of < 40. Extracts were tested in duplicate and considered positive when amplification was recorded in one or more test wells with a Ct value ≤40.

### PCR amplification and sequencing of *Bartonella ssr*A gene products

For sequencing, conventional PCR amplification of the *ssr*A gene was performed on all DNA extracts from both rodent spleens and fleas that were positive in the *ssr*A qPCR, based on a previously published protocol [20]. Each PCR reaction (25 μl) comprised 2.5 μl of PCR 10X buffer, 0.1μL Platinum Taq polymerase, 0.75μL MgCl_2_, 0.5 μl dNTPs (10 μM) (Invitrogen, USA) and 0.5 μl of each primer *ssr*A-F and *ssr*A-R at 10 μM [20]. Template DNA volume varied from 5–10 μl depending on *ssr*A assay Ct value. Nuclease-free water was used to make up the total reaction volumes as needed. Amplifications were performed with the following conditions: 94 °C for 2 minutes, followed by 40 cycles of 94 °C for 15 seconds, 60 °C for 60 seconds, and 72 °C for 30 seconds, and then a final extension step of 72 °C for 3 minutes. Positive and negative controls were included in each PCR run. PCR products were visualized by electrophoresis in a 1.5% agarose gel stained with GelRed (Cambridge Bioscience, Cambridge, UK). A sample was considered positive if a clearly defined DNA band of approximately 300 bp was visible in the gel and confirmed as *Bartonella* by sequencing of the product. To confirm and characterize the genotypes of *Bartonella* detected, amplicons with the expected size were purified using either a QIAquick PCR or Gel Extraction Purification Kit (Qiagen). Sequencing was performed at Source Biosciences (Nottingham, UK) using the same primers as for the detection PCR [33]. Sequence identity was confirmed using BLASTn, as implemented in the National Centre for Biotechnology Information (NCBI) web portal.

### PCR amplification and sequencing of *Bartonella glt*A gene products

Conventional PCR amplification of the *glt*A gene was performed on all rodent spleen DNA extracts, based on a previously published protocol [34]. Each PCR reaction (25 μl) comprised 12.5 μl of PCR 2X master mix (Promega, Madison, WI, USA), 1.25 μl 5% dimethyl sulphoxide (DMSO) (Sigma-Aldrich, St. Louis, USA), 1.25 μl molecular grade water (Qiagen), 2.5 μl of each oligonucleotide primer (10 μM), BhCS781.p (5ˈ-GGGGACCAGCTCATGGTGG-3ˈ) and BhCS1137.n (5ˈ- AATGCAAAAAGAACAGTAAACA-3ˈ) [34], and 5 μl DNA template. Amplifications were performed on a PTC-240 DNA-Engine (MJ Research/BioRad Technologies, USA) with the following conditions: 94 °C for 2 minutes, followed by 40 cycles of 94 °C for 30 seconds, 54.3 °C for 30 seconds, and 72 °C for 2 minutes, then a final step of 72 °C for 7 minutes. Positive and negative controls were included in each PCR run. PCR products were visualized by electrophoresis in a 1.5% agarose gel stained with ethidium bromide (Invitrogen, USA). A sample was considered positive if a clearly defined DNA band of approximately 379 bp was visible in the gel and confirmed as *Bartonella* by sequencing of the product. Purification, sequencing and BLAST analyses were conducted as for the *ssr*A gene.

### Phylogenetic analyses for *ssr*A and *glt*A gene products

**Fig 2:**
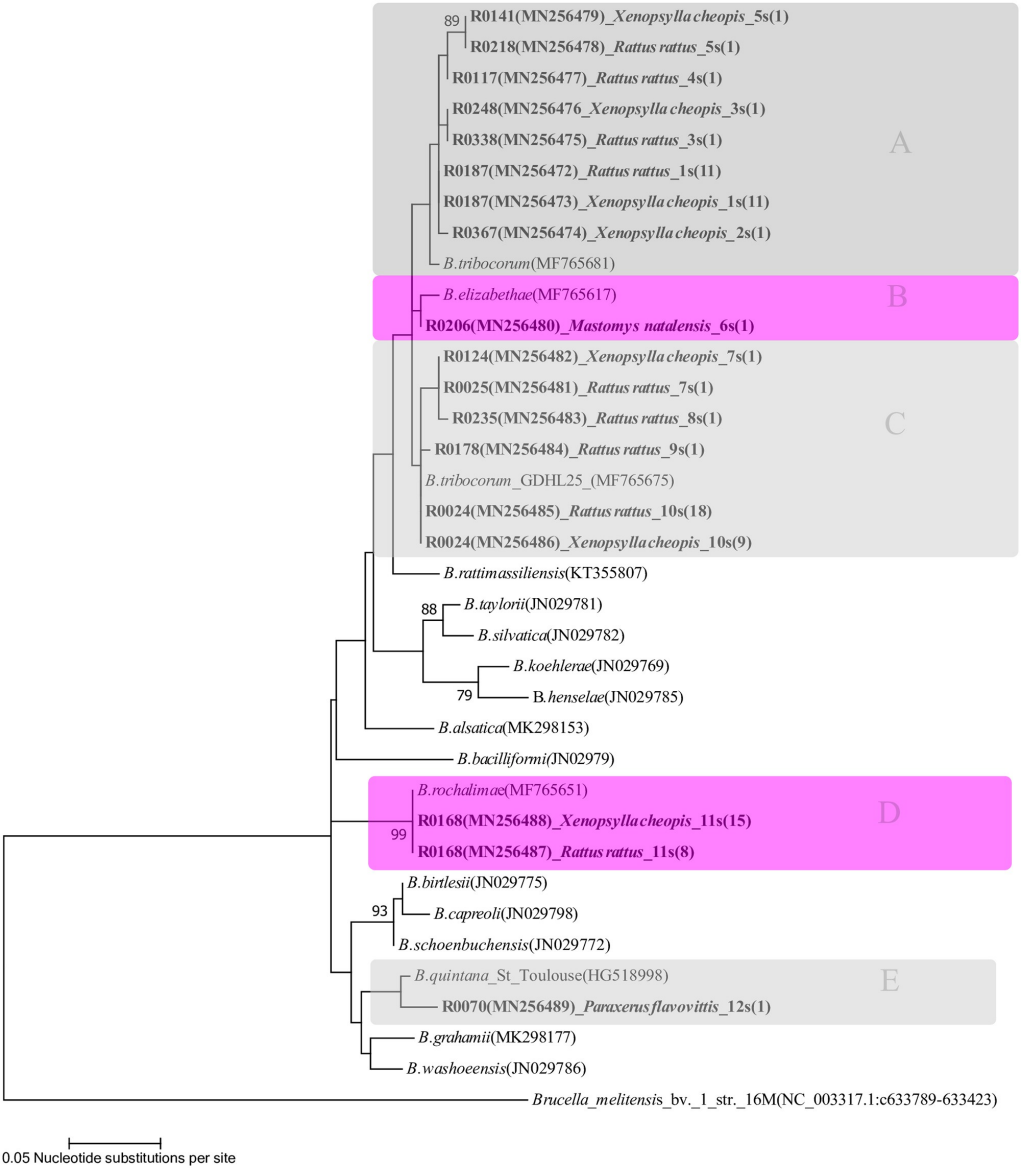
Phylogenetic tree showing the relatedness of the *Bartonella ssr*A gene sequences (237bp gene fragments) derived from 45 rodent spleen tissue samples (43 *R*. *rattus*, 1 *M*. *natalensis* and 1 *P*. *flavovottis)* and 39 *X*. *cheopis fleas* collected in northern Tanzania. A single representative sample sequence is included for each combination of *Bartonella* genotype identified in this study and host of origin. Genotypes (1s–12s) and groups (As–Es) are indicated by lettering. Groups A, C and E are shaded grey, with groups B and D in pink. The phylogenetic tree was constructed using the maximum likelihood method based on a Kimura 2-parameter substitution model [41], as determined by Model test as implemented in MEGA 7.0 [35]. The tree with the highest log likelihood is shown and drawn to scale, with branch lengths shown in terms of the number of substitutions per site. Vertical branches indicate identical sequences. The numbers at the nodes correspond to bootstrap values higher than 70% after 1000 replicates. Sequences from this study are labelled with unique identifiers, with prefix “R” followed by sample identifier numbers, Genbank accession number, the rodent or flea host species, the genotype code and the number of samples yielding each genotype (in parentheses). Sequences from reference strains of *Bartonella* are included with the *Bartonella* species name and GenBank accession numbers given in parentheses. *Brucella melitensis* was included as an outgroup.

**Fig 3:**
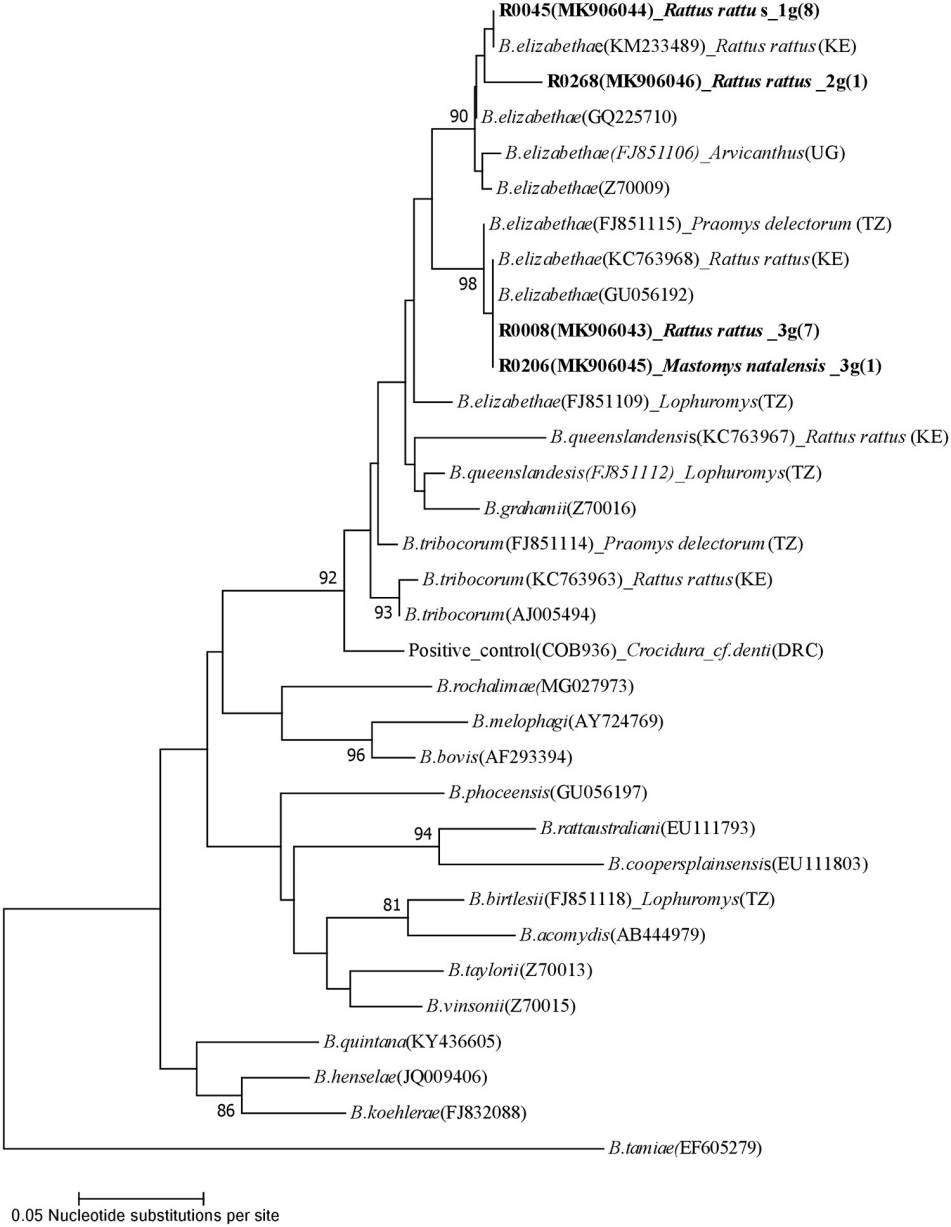
Phylogenetic tree showing the relatedness of the *Bartonella glt*A gene sequences (283bp fragments) derived from 17 spleen tissue samples from rodents (16 *R*. *rattus* and 1 *Mastomys natalensis)* trapped in northern Tanzania. A single representative sample sequence is included for each genotype identified in this study, with the exception of genotype 3g to illustrate the identical sequences obtained from *R*. *rattus* and *M*. *natalensis*. Sequences from this study are labelled with unique identifiers, with prefix “R” followed by sample identifier numbers, Genbank accession number, the rodent or flea host species, the genotype code and the number of samples yielding each genotype (in parentheses). Reference *Bartonella* sequences from rodents trapped elsewhere in East Africa obtained from GenBank are indicated by GenBank accession numbers in parentheses, rodent species and country code (Kenya (KE) [22], Uganda (UG) [26] Tanzania (TZ) [25], Democratic Republic of Congo (DRC) [25]). The sequence obtained for the known *Bartonella* positive control sample provided by a colleague from a previous study is included and indicated with a unique identification number (COB936) [24].

Incomplete and poor quality sequences (e.g. with ambiguous peaks) were excluded from phylogenetic analysis for both *ssr*A and *glt*A gene fragments. For each gene, sequences were aligned using the ClustalW algorithm, implemented in MEGA 7.0 [35]. The model test function in MEGA 7.0 was used to select the best-fitting nucleotide substitution models, which were then incorporated into a phylogenetic analysis based on a maximum likelihood optimality criterion for tree reconstruction, with 1000 bootstrap pseudoreplicates. For the *ssr*A analysis, rodent spleen and flea sequences from this study were aligned with reference *ssr*A sequences from cultured *Bartonella* species downloaded from GenBank (see GenBank accession numbers in Fig 2). A *Brucella melitensis* sequence was used as the outgroup [36]. For analysis of the *glt*A data, sequences from study rodent spleens were aligned with those from *Bartonella* reference strains obtained from GenBank and also with representative sequences from previous studies conducted in East Africa (see GenBank accession numbers in Fig 3). Reference sequences in the alignment included *glt*A sequences from previous studies of *Bartonella* in rodents from Tanzania [25], Kenya [22], the Democratic Republic of Congo [24,25] and Uganda [26] that included either similar rodent species or *Bartonella glt*A sequences similar to those obtained in this study. A *B*. *tamiae glt*A sequence obtained from an African bat was used as the outgroup [37].

### Statistical analyses

Statistical analyses were performed in R [38]. Exact binomial proportions and confidence intervals for prevalence estimates were calculated using the package ‘binom’ [39]. Generalized linear mixed models (GLMM), with binomial family and logit link function, were used to examine variables associated with rodent and flea *ssr*A qPCR test status (qPCR positive vs negative) and implemented using the package ‘lme4’ [40]. For rodents, the dataset for these analyses was limited to *R*. *rattus* only, given the dominance of this species. Explanatory variables considered in the GLMM for rodent *ssr*A qPCR status included host sex and reproductive maturity (mature or immature), which were determined based on external sexual characteristics [30]. Trapping season (wet or dry), trapping district (Moshi Municipal or Moshi Rural) and rodent abundance were also included as explanatory variables for analysis. Adjusted trap success data were calculated by dividing the total number (n) of rodents caught per village by the corrected number of trap nights, which is calculated as: total number of trap nights (number of traps x number of nights) minus lost trap nights (sum of number of closed, damaged or lost traps / 2) and expressed as a percentage [29]. The village identification variable was included as a random effect to account for the clustered sampling strategy. For fleas, rodent *ssr*A qPCR test status was the only explanatory variable evaluated in the GLMM and the village identification variable was included as a random effect. Initial maximal multivariable models were created including all candidate explanatory variables and likelihood ratio tests were used to compare candidate models and guide model selection.

## Results

### Sample characteristics

Spleen samples from a total of 381 rodents were available for testing. The majority (n = 317, 83.2%), were from black rats (*R*. *rattus*) (Table 1). Other rodent species tested included: house mice (*Mus musculus*, n = 44, 11.5%); African pygmy mice (*Mus minutoides*, n = 3, 0.8%); multimammate mice (*Mastomys natalensis*, n = 8; 2.1%), spiny mice (*Acomys wilsonii*, n = 6, 1.6%) and striped bush squirrels (*Paraxerus flavovittis*, n = 3, 0.8%). Of the rodents tested, 219 individuals (57.5%) were female. The majority of all sampled rodents (n = 224, 58.8%) were classified as sexually mature based on external sexual characteristics. A total of 265 of the 381 rodents (69.6%) were trapped during wet season sampling. A total of 513 fleas were collected from 153 of the 381 (40.2%) rodents included in the study. Flea species identified were *Xenopsylla cheopis* (n = 306), *Echidnophaga gallinacea* (n = 204) and *Ctenocephalides felis* (n = 3). DNA extracts from a total of 193 *Xenopsylla cheopis* collected from 124 rodents (*Rattus rattus* n = 118, *Mus musculus* n = 4 and *Mastomys natalensis* n = 2) were available for *Bartonella* testing using *ssr*A.

**Table 1:** Summary of rodent species and their *Bartonella* detection status defined by *ssr*A qPCR and sequence confirmed by *glt*A PCR testing of spleen samples.

Rodent species	Number of spleen samples tested	*Bartonella ssr*A qPCR Positive n (%)	*Bartonella ssr*A qPCR Ct values of Positives	*Bartonella glt*A Product Sequence Positive n (%)
*Acomys wilsonii*	6	0 (0)	NA	0 (0)
*Mastomys natalensis*	8	2 (25.0)	33.23 & 33.54	1 (12.5)
*Mus minutoides*	3	0 (0)	NA	0 (0)
*Mus musculus*	44	0 (0)	NA	0 (0)
*Paraxerus flavovottis*	3	1 (33.3)	36.49	0 (0)
*Rattus rattus*	317	54 (17.0)	median value of 33.07, range 24.25–39.56	16 (5.0)
**Total**	**381**	**57 (15.0)**		**17 (4.4)**

### *Bartonella* detection in rodent spleens and risk factors for rodent infection

*Bartonella* DNA was detected by *ssr*A qPCR in a total of 57 of 381 (15.0%, 95% CI 11.5–18.9%) rodents screened (Table 1). Samples derived from *Mastomys natalensis*, *Paraxerus flavovittis* and *R*. *rattus* (Table 1) were all classified as *Bartonella* positive by this test. The positive control used in *ssr*A qPCR runs to test rodent spleen extracts had a mean Ct value of 32. The assay showed a 100% lower limit of detection of 1.8 fg of *Bartonella quintana* DNA control. For the logistic regression analysis considering data from *R*. *rattus* only, rodent reproductive maturity status was the only significant risk factor (LRT: *χ*_*2*_ = 13.30, df = 1, *p* < 0.0003), with reproductively mature individuals more likely to be *ssr*A qPCR positive (OR 3.42, 95% CI 1.69–6.89, *p* < 0.001). None of the other candidate variables evaluated (rodent sex, trapping season, trapping district or rodent abundance at trapping village) were significantly associated with *R*. *rattus ssr*A qPCR test status. The breakdown of rodents trapped by village is given in Table 2.

**Table 2:** Summary of rodent trapping data and the *Bartonella ssr*A and *glt*A genotypes detected in trapped rodents by village around Moshi, Tanzania.

Village code	District	Total number of rodents tested for *Bartonella*	Adjusted trap success [29]	*Bartonella* genotypes identified, with data on the number of individuals and rodent species each genotype was detected in	
				*ssr*A genotypes	*gltA* genotypes
A	Rural	12	9.79	1s–2 * *Rattus rattus*	3g–2 * *Rattus rattus*
B	Rural	13	4.28	7s–1 * *Rattus rattus* 10s–1 * *Rattus rattus*	-
C	Municipal	31	4.77	10s–1 * *Rattus rattus*	1g–1 * *Rattus rattus*
D	Municipal	25	2.68	10s–1 * *Rattus rattus* 2s–1 * *Paraxerus flavovittis*	1g–1 * *Rattus rattus*
E	Rural	39	5.28	4s–1 * *Rattus rattus* 10s–2 * *Rattus rattus*	1g–1 * *Rattus rattus* 3g–1 * *Rattus rattus*
F	Municipal	76	10.8	1s–5 * *Rattus rattus* 9s–1 * *Rattus rattus* 10s–1 * *Rattus rattus* 11s–5 * *Rattus rattus*	1g–1 * *Rattus rattus* 3g–3 * *Rattus rattus*
F (visit 2)	Municipal	33	4.42	1s–1 * *Rattus rattus* 11s–3 * *Rattus rattus*	
G	Rural	15	1.94	6s–1 * *M*. *natalensis*	3g–1 * *M*. *natalensis*
H	Rural	35	4.69	1s–1 * *Rattus rattus* 5s–5 * *Rattus rattus* 8s–1 * *Rattus rattus* 10s–3 * *Rattus rattus*	1g–2 * *Rattus rattus* 3g–1 * *Rattus rattus*
J	Rural	19	2.70	10s–4 * *Rattus rattus*	2g–1 * *Rattus rattus*
K	Municipal	23	3.19	10s–1 * *Rattus rattus*	-
L	Rural	22	2.93	1s–2 * *Rattus rattus* 10s–3 * *Rattus rattus*	1g–2 * *Rattus rattus*
M	Municipal	38	5.06	3s–1 * *Rattus rattus*	

Village codes correspond to village locations indicated in Fig 1. Distinct *ssr*A and *glt*A genotypes are identified by a sequential number and “s” or “g” respectively. Data on trap success are as reported in [29]

### *Bartonella* detection in fleas

*Bartonella* DNA was detected by *ssr*A qPCR in 53 of 193 (27.5%, 95% CI 21.3–34.3%) *X*. *cheopis* flea extracts. All *ssr*A qPCR positive flea extracts were collected from *R*. *rattus* individuals. The positive control used in *ssr*A qPCR runs to test flea extracts had a mean Ct value of 32. Logistic regression analysis identified a relationship between flea *ssr*A qPCR test status and the *ssr*A qPCR test status of the rodent that each flea was collected from (LRT: *χ*_*2*_ = 20.73, df = 1, *p* < 0.001). *X*. *cheopis* fleas collected from *ssr*A qPCR positive rodents were more likely to themselves be *ssr*A qPCR positive (OR 7.23, 95% CI 2.90–17.97, *p* < 0.001).

### Characterisation of *Bartonella* in Tanzanian rodents and fleas by *ssr*A sequencing

Full length sequences of the *ssr*A gene target were obtained from 45 of 57 rodents spleen samples (*Rattus rattus* n = 43, *Mastomys natalensis* n = 1 and *Paraxerus flavovottis* n = 1) and 39 of 53 *ssr*A positive *X*. *cheopis* fleas. From the 237bp *ssr*A sequences amplified, 12 unique genotypes (1s to 12s) were identified in the sequences from rodent and flea populations combined (Table 3; Fig 2). The 12 genotypes were grouped into monophyletic groups with ≥97% similarity within the group. The following groupings were identified: 1) group As: Genotypes 1s to 5s clustering with a *B*. *tribocorum* reference sequence from strain GDHL73 (GenBank accession number MF765681); 2) group Bs: genotype 6s was unique but clustered with a *B*. *elizabethae* reference sequence (GenBank accession number MF765617); 3) group Cs: genotypes 7s to 10s clustering with a sequence from *B*. *tribocorum* strain GDHL25 (GenBank accession number MF765675); 4) group Ds: genotype 11s clustering with a *B*. *rochalimae* reference sequence (GenBank accession number MF7651); and 5) group Es: genotype 12s from *P*. *flavovittis*, which was mostly closely related to a *B*. *quintana* reference sequence (GenBank accession number HG518998). The distribution of *ssr*A genotypes between trapping villages (Table 2) show that more frequently detected *ssr*A genotypes (1s, 10s and 11s) were present in rodents trapped at multiple villages and provides no evidence of spatial segregation of the genotypes in this rodent population. Data on *ssr*A genotypes were available for fourteen pairs of *X*. *cheopis* fleas and *R*. *rattus* hosts (n = 11 *R*. *rattus* including three from which two *X*. *cheopis* were collected and tested). For seven pairs the *ssr*A genotype detected in fleas and rodent hosts were identical, but in the other seven pairs the genotypes differed. At the group level, 11 flea and host pairs had sequences from the same *ssr*A group and three pairs differed.

**Table 3:** Summary of *Bartonella ssr*A genotypes identified in rodent spleens and fleas from Moshi, Tanzania.

*ssr*A genotypes	*ssr*A Group	Rodent species and number of positive samples	Flea species and number of positive samples	Closest *Bartonella* species (GenBank ID)^a^	% similarity to closest *Bartonella* spp. (number of base pair identities/ total base pair length)
1s	As	11 * *R*. *rattus* (MN25672)	11 * *X*. *cheopis* (MN25673)	*B*.*tribocorum* (MF765681)	99 (240/244)
2s	As	-	1 * *X*. *cheopis* (MN25674)	*B*.*tribocorum* (MF765681)	99 (217/222)
3s	As	1 * *R*. *rattus* (MN25675)	1 * *X*. *cheopis* (MN25676)	*B*.*tribocorum* (MF765681)	99 (228/233)
4s	As	1 * *R*. *rattus* (MN25677)	-	*B*.*tribocorum* (MF765681)	99 (239/244)
5s	As	1 * *R*. *rattus* (MN25678)	1 * *X*. *cheopis* (MN25679)	*B*.*tribocorum* (MF765681)	98 (237/244)
6s	Bs	1* *M*. *natalensis* (MN25680)	-	*B*.*elizabethae* (MF765617)	99 (222/224)
7s	Cs	1 * *R*. *rattus* (MN25681)	1 * *X*. *cheopis* (MN25682)	*B*.*tribocorum* (MF765675)	99 (242/244)
8s	Cs	1 * *R*. *rattus* (MN25683)	-	*B*.*tribocorum* (MF765675)	99 (239/244)
9s	Cs	1 * *R*. *rattus* (MN25684)	-	*B*.*tribocorum* (MF765675)	99 (236/237)
10s	Cs	18 * *R*. *rattus* (MN25685)	9 * *X*. *cheopis* (MN25686)	*B*.*tribocorum* (MF765675)	100 (244/244)
11s	Ds	8 * *R*. *rattus* (MN25687)	15 * *X*. *cheopis* (MN25688)	*B*.*rochalimae* (MF765651)	100 (246/246)
12s	Es	1* *P*. *flavovottis* (MN25689)	-	*B*.*quintana* (HG518998)	98 (233/239)

The number of individuals of each rodents (n = 45) and fleas (n = 39) species from which each genotype was obtained are shown, as well as data on % similarity to reference *Bartonella* species sequences, with the number of base pair identities indicated in parentheses. The Genbank accession numbers for each genotype are also indicated in parentheses in columns 2 and 3.

^a^ The closest reference sequences to the study sequences were selected from fully characterized sequences in Genbank obtained from cultures.

### Characterisation of *Bartonella* in Tanzanian rodents by *glt*A sequencing

Full length sequences of the *glt*A gene were obtained from 17 rodent spleen DNA extracts (*R*. *rattus* n = 16 and *M*. *natalensis* n = 1). To be conservative, the alignment was pruned to the length of the shortest sequence (283 bp) and three unique genotypes were identified in this fragment (Fig 3). The correspondence between *glt*A and *ssr*A genotypes and groups is shown in Table 4. Only one *R*.*rattus* individual had a *glt*A sequence without a corresponding *ssr*A sequence. For the 16 remaining *glt*A genotypes, the same individuals all yielded *ssr*A sequences falling into groups As to Cs (Table 4). Eight sequences (genotype 1g, GenBank accession number MK906044) collected from *R*. *rattus* were identical to a *B*. *elizabethae* sequence obtained from a *R*. *rattus* sampled previously at a rural site in Kenya (strain B29297 [22], GenBank accession number KM233489). A second genotype (2g, GenBank accession number MK906046) was identified in one *R*. *rattus* and showed and 97% similarity (278 of 284 base pair matches) to a *B*.*elizabethae* sequence (GQ225710). Eight sequences (genotype 3g) collected from *M*. *natalensis* (GenBank accession number MK906045) and from *R*. *rattus* (GenBank accession number MK906043) were identical to a sample identified in a *R*. *rattus* from an urban site in Kenya (strain B28391 [22], GenBank accession number: KC763968) and to a cultured reference strain of *B*. *elizabethae* (strain BR02, GenBank accession number: GU056192).

**Table 4:** Summary of *glt*A genotypes identified in rodent spleens (n = 17) from Moshi, Tanzania and the correspondence with *ssr*A genotypes identified in the same species and individuals. Data on the % similarity to reference *Bartonella* species sequences, and the number of base pair identities are indicated. The Genbank accession numbers for each genotype identified in the study are indicated.* The closest reference sequences to the study sequences were selected from fully characterized sequences in Genbank obtained from cultures.

*glt*A Genotype	GenBank accession number	Rodent species and number of positive samples	Closest *Bartonella* species (GenBank ID)*.	% similarity to closest *Bartonella* spp. (number of base pair identities/ total base pair length)	*ssr*A group and genotype
1g	MK906044	8 * *R*. *rattus*	*B*.*elizabethae*(GQ225710)	99.65 (282/283)	Group C:10s
2g	MK906046	1 * *R*. *rattus*	*B*.*elizabethae* (GQ225710)	97 (278/284)	Group C:10s
3g	MK906043	5 * *R*. *rattus*	*B*.*elizabethae*(GU056192)	100 (283/283)	Group A: 1s
3g	MK906043	1 * *R*. *rattus*	*B*.*elizabethae*(GU056192)	100 (283/283)	Group A: 4s
3g	MK906045	1 * *M*. *natalensis*	*B*.*elizabethae*(GU056192)	100 (283/283)	Group B: 6s
3g	MK906043	1 * *R*. *rattus*	*B*.*elizabethae*(GU056192)	100 (283/283)	No *ssr*A typing obtained

## Discussion

This study reveals substantial variation in *Bartonella* genotypes among rodents and their fleas in a previously uncharacterised region of northern Tanzania (the Moshi Municipal and Moshi Rural Districts). *Rattus rattus* was the most common rodent species trapped and showed a high *Bartonella* prevalence defined by *ssr*A qPCR, which is consistent with the global distribution of *Bartonella* species in *Rattus* [42]. Within *R*. *rattus*, the probability of qPCR positivity was higher in reproductively mature individuals as compared to immature individuals, consistent with other studies (performed in the USA) that have found an association with age [43,44]. Sequencing of *ssr*A and *glt*A gene fragments revealed a variety of genotypes and the majority of sequences obtained showed greatest similarity to *B*. *tribocorum* and *B*. *elizabethae* reference sequences, both of which have been isolated in humans with febrile illness [6]. Sequences similar to *B*. *rochalimae* and *B*. *quintana* were also identified based on *ssr*A sequencing. These species were not detected by sequencing of the *glt*A, indicating reduced sensitivity of the *glt*A conventional PCR for detection of *Bartonella* species as compared to the *ssr*A qPCR. This is consistent with the findings of a previous study [20].

The *ssr*A qPCR was used to estimate prevalence in rodents and fleas, and the sequencing of *ssr*A and *glt*A PCR products to assess genetic variation and characterise the *Bartonella* detected. The overall prevalence of *Bartonella* (15%) detected in rodents using the *ssr*A qPCR was lower than has been detected in many comparable studies of global rodent populations [21,45], including studies that have used a less sensitive *glt*A assay for prevalence determination [46,47]. In a previous Tanzanian study of indigenous rodent species an overall *glt*A prevalence of 41% was detected [25]. A Ugandan survey using *glt*A to test invasive and indigenous rodent populations found variable prevalence across species, with higher prevalence in indigenous species (60% in *Arvicanthus niloticus* and 61% in *Cricetomys gambianus*), but low prevalence (1.4%) was recorded in invasive *R*. *rattus* [26]. Similarly, in Kenya the *Bartonella* prevalence determined by culture varied by rodent species [22]. Considering the data from *R*. *rattus* only, the prevalence seen in this study and previous African studies reveals consistently lower prevalence in comparison to *R*. *rattus* sampled in Asia and tested using *ssr*A qPCR methods (e.g. 32.5% [48]). The prevalence of *Bartonella* detection in *X*. *cheopis* fleas in this study using the *ssr*A qPCR was also lower than has been recorded in this species in the USA, where 190 of 200 (95%) *X*. *cheopis* tested were positive for Bartonella DNA [32]. The low prevalence of *Bartonella* in *R*. *rattus* and *X*. *cheopis* observed in this study are consistent with several other studies conducted in Africa. It has been argued that this pattern of lower *Bartonella* prevalence in African *R*. *rattus* populations could be attributed to host escape during colonization [49]. Further investigation of native and invasive rodent populations across Africa would be needed to investigate this further, and also evaluate the possible implications for human disease risk on the continent.

Phylogenetic analysis of sequences from rodents and their fleas revealed high concordance of sequences between hosts and ectoparasites. Overall, 10 distinct *ssr*A genotypes were identified that were most similar to reference sequences of *B*. *tribocorum* and *B*. *elizabethae* (Groups As to Cs), with only one genotype (10s) showing an identical match to a published *B*. *tribocorum* sequence in GenBank. However, since all of the reference sequences that were most similar were from a single study in China [50] it is important to recognise the limited reference data available currently and need for future comparison to datasets from other geographic areas to further evaluate these data on the diversity and types of *Bartonella* found in Tanzania, Moreover, *B*. *elizabethae* and *B*. *tribocorum* share identical published *ssr*A sequences in Genbank and our results show clustering in the phylogenetic tree (Fig 2), so the two species cannot be distinguished by this *ssr*A fragment. The other two *Bartonella* species identified were: 1) a single sequence (11s) with greatest similarity (98%) to *B*. *quintana* obtained from a sample from a *P*. *flavovittis* host; and 2) a sequence obtained from multiple samples of *R*. *rattus* and *X*. *cheopis* that showed an identical match to *B*. *rochalimae*, emphasising the diversity of *Bartonella* present in rodents in Tanzania. To the best of our knowledge, this is the first report of molecular detection and characterization of *Bartonella* species in rodents and their associated ectoparasites in Africa using the *ssr*A gene target. The scope for comparison with other sequences is thus limited, as there is currently little reference material on *ssr*A sequences from *Bartonella* sampled elsewhere, particularly in Africa.

In contrast, the *glt*A gene has been widely used to study *Bartonella* globally. Phylogenetic analysis of sequences from 17 *glt*A PCR products from this Tanzanian rodent population (16 *R*. *rattus* and 1 *M*. *natalensis*) showed the highest similarity to reference sequences of *B*. *elizabethae*, which has multiple published sequences in Genbank, including many from east Africa. The association of sequences similar to *B*. *elizabethae* with *Rattus* spp. in this study is consistent with similar findings from Malaysia [51] and Thailand [48]. *B*. *elizabethae* has also been identified in different rodent species in Africa [25,26]. Identical *glt*A sequences were amplified from *R*. *rattus* and *M*. *natalensis* in our study, suggesting possible transmission between different rodent species in the Tanzania site or a shared common source of infection. Identical sequences were also identified previously in *R*. *rattus* sampled at both rural (strain B28297, accession number KM233489, from Asembo, Kenya) and urban (strain B28391, accession number KC763968, from Kibera, Nairobi Kenya) sites in Kenya [22]. This suggests that similar *Bartonella* could be found in rodents in Kenya and Tanzania. However, these comparisons are based on short sequences of a single gene target only. Longer sequences from multiple genes and greater sampling effort across the region would be required to robustly confirm sharing of genotypes to trace source populations or determine patterns of host connectivity

Several studies of zoonotic disease have shown that a variety of pathogens account for high proportions of febrile illness in northern Tanzania but that considerable proportions remain unexplained [16,52]. *Bartonella* species have been identified as important causes of human febrile illness in several global settings but there has been little investigation of the impact of bartonellosis upon human health, in Africa particularly. The finding of *Bartonella* genotypes that are most similar to *B*. *elizabethae*, *B*. *rochalimae* and *B*. *quintana* reference sequences in rodents trapped in and around households in Moshi, Tanzania, and the fleas collected from these rodents, indicates the possibility that *Bartonella* infection may be responsible for an as yet unknown proportion of febrile illnesses in this region. Efforts are needed to determine the clinical impact of bartonellosis in this region and increase awareness about *Bartonella* and other zoonotic pathogens among physicians and health care workers, especially where the cause of large proportions of febrile illness remains unknown. Our results also demonstrate that molecular detection tools can be effectively used for surveillance and diagnostic of zoonotic pathogens in resource limited settings.
